# Giant Perineal Leiomyoma: A Case Report and Review of the Literature

**DOI:** 10.1155/2014/629672

**Published:** 2014-06-04

**Authors:** Wolf von-Waagner, Huifei Liu, Antonio I. Picon

**Affiliations:** ^1^Department of Surgery, Division of Surgical Oncology, Staten Island University Hospital, 2nd Floor, 256B Mason Avenue, Staten Island, NY 10305, USA; ^2^Department of Pathology, Staten Island University Hospital, 475 Seaview Avenue, Staten Island, NY 10305, USA

## Abstract

We report the case of a 40-year-old woman who presented with a large perineal mass with no rectal or vaginal involvement. Imaging could not rule out malignancy. She underwent wide surgical excision. Histological analysis revealed a large atypical leiomyoma, measuring 24 × 12 × 8 cm. Followup after two years showed no recurrence and she has been asymptomatic since surgery. This is the largest perineal leiomyoma reported so far.

## 1. Introduction

Soft tissue tumors include any nonepithelial tissue other than bone, cartilage, central nervous system, hematopoietic, and lymphoid tissues. Leiomyomas are benign soft tissue tumors. Leiomyomas are common tumors of mesenchymal origin, are well-circumscribed neoplasms, and can develop where smooth muscle is present. Leiomyomas are encountered most commonly in the uterus and the skin. Leiomyomas are divided into two groups as superficial and deep. Perineal-genital leiomyomas are considered to be of superficial location. External genital soft tissue leiomyomas are extremely rare [1]. Perineal leiomyomas are extremely rare monoclonal mesenchymal tumors, with an incidence of 3.8% of all benign soft tissue tumors [2]. We report a case of a giant perineal leiomyoma.

## 2. Case Report

**Figure 1:**
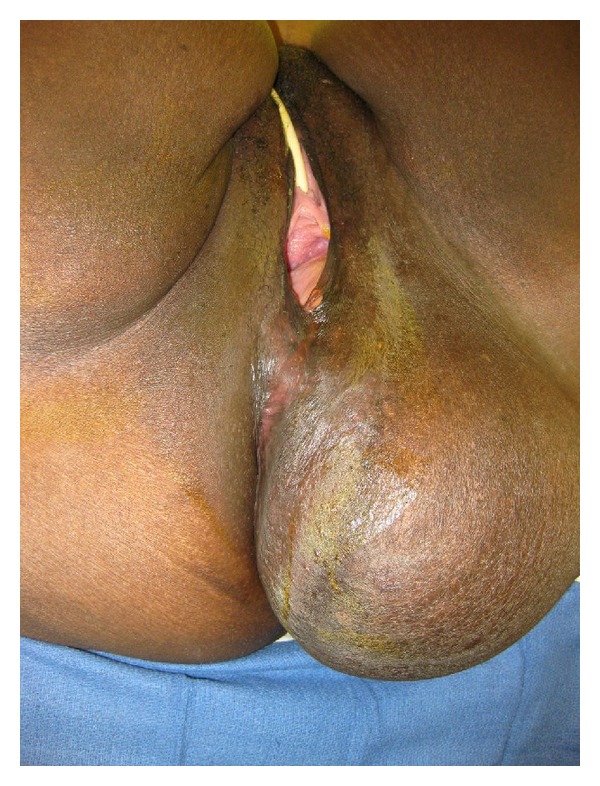
Left perineal mass. Examination of the vagina reveals a bulging mass through the left wall of the vagina but without any direct invasion. Examination of the rectum revealed normal tone with displacement of the left lateral rectal wall towards the midline.

A 40-year-old African American, morbidly obese female patient was referred to our service for evaluation of left buttock mass (Figure 1). She reported minor discomfort and she did not tolerate sitting down for long periods of time. She described that the mass had been growing slowly for the past six months. She described no urinary, rectal, or gynecological symptoms. She had no prior surgeries. On physical examination there was a bulging mass on the left lateral wall of the vagina without any direct invasion; the rectum had a normal tone with displacement of the left lateral wall towards the midline; the left perineal area revealed a 25 × 14 cm soft mass.

**Figure 2:**
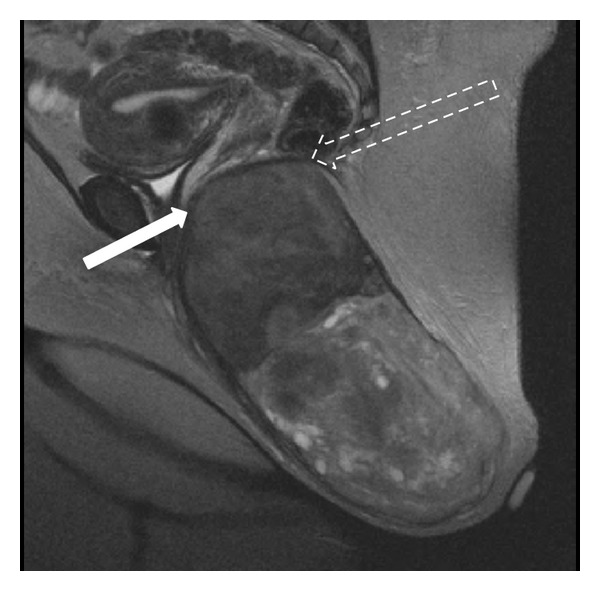
MRI sagittal plane. 23 cm (craniocaudal) mass exhibits midrange T2 weighted signal superiorly with diminished T2 weighted signal at its inferior position. White arrow showing the mass abutting the posterior wall of the vagina. Dashed arrow showing the rectum wall.

Endoscopic ultrasound demonstrated a demarcated fat plane between the rectum and the mass. However, the CT scan found no clear fat plane between the mass and the surrounding structures. Imaging revealed the mass within the subcutaneous tissue of the left perirectal region, displacing the anal canal to the right and displacing superiorly the levator ani musculature. Uterine leiomyomas and diverticulosis were found incidentally. The MRI dimensions of the mass were 23 × 8 × 11 cm and showed no muscular or osseous invasion, with well-defined walls and considerable enhancement with a preliminary diagnosis of a pedunculated leiomyoma; however, a soft tissue sarcoma could not be ruled out (Figure 2). There was concern that this tumor was directly invading the surrounding structures, such as the rectum, vagina, anal sphincter, or adnexa. On the basis of the results of the imaging studies, a large leiomyoma was suspected, but we could not rule out a low grade soft tissue sarcoma. Surgical resection was performed and no direct extension into surrounding structures was found; muscle fibers of the anal sphincter were densely adherent to the tumor and were divided close to the tumor in order to preserve function. After the surgery the patient had no anal dysfunction and she was discharged just with pain medication. She denied fecal incontinence or dyspareunia. After two-year followup she remains without recurrence.

## 3. Pathology

**Figure 3:**
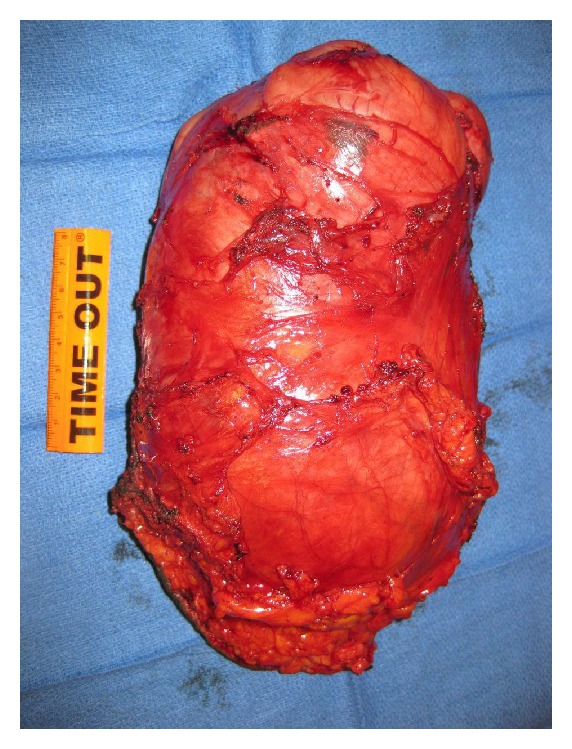
Resected mass measuring 24 × 12 × 8 cm with smooth tan-pink surface and weighting 1660 gm. On sections, the mass shows areas of smooth, whorled tan-white tissue, with areas of cystic degeneration, edema, and rare slightly softer, white opaque areas. No hemorrhage or necrosis is identified grossly.

**Figure 4:**
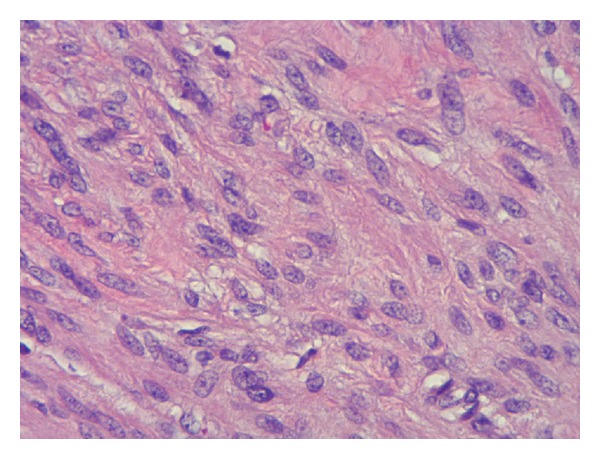
Hematoxylin-eosin staining of tumor section showing well-differentiated smooth muscle cells with eosinophilic cytoplasm and bland, blunt-ended nuclei (40x).

**Figure 5:**
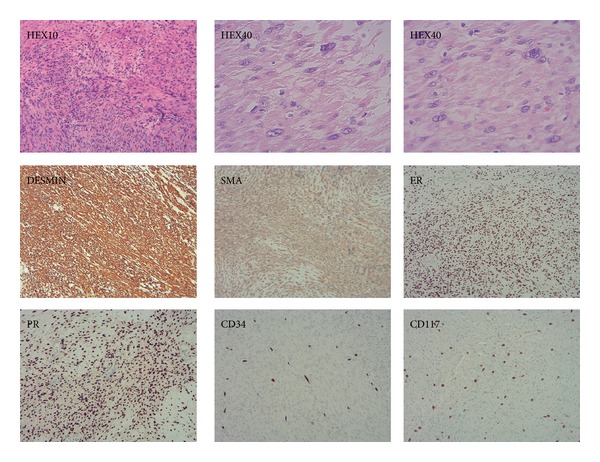
Morphology and Immunohistochemical staining of tumor; HE: hematoxylin-eosin, HEX10 (low power field), HEX40 (high power field), Desmin (positive), SMA: smooth muscle actin (positive), ER: estrogen receptor (positive), PR: progesterone receptor (positive), CD34 (negative), and CD117 (negative).

During surgery, an ellipsoid well-circumscribed mass measuring 24 × 12 × 8 cm was noted without invasion to surrounding tissue (Figure 3). Intraoperative consultation was performed. The cut surface of the mass was smooth, tan-white, whorled pattern, with areas of cystic degeneration and edema. No hemorrhage and necrosis were identified grossly. Frozen section assessment was reported as spindle cell lesion, favor smooth muscle neoplasm. Histopathology of permanent sections showed well-differentiated spindle cells arranged in orderly intersecting fascicles. These cells had eosinophilic cytoplasm and mostly bland, uniform, cigar-shaped nuclei, resembling normal smooth muscle cells (Figure 4). There were several areas showing moderate cellular atypia. Immunohistochemistry showed that these tumor cells were positive for desmin, smooth muscle actin, estrogen receptor, and progesterone receptor and negative for S100, CD117, and CD34, confirming the smooth muscle origin of the tumor (Figure 5). No coagulative tumor cell necrosis was identified. The highest mitotic figures were three mitoses per 10 high power fields. Final diagnosis was atypical leiomyoma with low risk of recurrence.

## 4. Methods

The literature review for this case encompasses articles from Pubmed using the key words, perineal leiomyoma, extrauterine smooth muscle tumor, and vulvar leiomyoma.

We also performed an extended search in the grey literature. This is the largest perineal leiomyoma reported so far.

## 5. Discussion

The differential diagnosis on perineal masses can be challenging due to the wide variety of tissues in the pelvic and perineal area (Table 1). In this particular case, based on clinical findings and imaging, our primary diagnosis was a large leiomyoma. The lack of local invasion into the surrounding tissues and the mobility of the mass pointed toward a more benign tumor rather than a low grade soft tissue sarcoma. However, due to the size and location of the tumor, a low grade sarcoma was in the differential diagnosis [3]. We decided to perform a wide surgical resection [3, 4]. During the resection the mass did not have direct extension into the rectum or the vagina. After the resection the sphincter mechanism was noted to be intact.

The published literature regarding patients with perineal leiomyoma is scarce [5]. There are few case reports published and all of them are of women of childbearing age presenting with painless perineal masses. These cases usually involve a much smaller mass with an average size of 5 cm, and the majority are associated with uterine leiomyomatosis [3, 6] (Table 2).

Among the imaging studies, magnetic resonance imaging with intravenous contrast (MRI) is an excellent imaging tool for the characterization and diagnosis of perineal soft tissue lesions. However, perineal leiomyomas can have variable MRI presentations [1, 7] (Table 3). It is important to consider a gastrointestinal stromal tumor (GIST) as a differential and distinguish it from a leiomyosarcoma [1]. Distinguishing leiomyomas from their malignant variant leiomyosarcomas can be a challenge from the imaging point of view, because some benign-appearing lesions behave aggressively with time [8]. Leiomyosarcomas usually present as nonspecific soft tissue masses with low-intermediate signal intensity on T1 weighted images and high signal intensity on T2 weighted images [9]. Leiomyosarcomas are mostly irregular, poorly defined tumors without a limiting membrane. Large masses usually show central necrosis and a thick, irregular rim-like enhancement after contrast administration on MRI, with bone involvement in 10% of cases [9]. Unfortunately, imaging appearances are nonspecific and cannot be differentiated from other aggressive nonfatty lesions [10].

Rectal GIST typically are heterogeneously enhancing exophytic masses that can sometimes present with hemorrhage and although the radiologic features of GIST are often distinct from those of epithelial tumors, criteria to separate GIST radiologically from other nonepithelial tumors have not yet been fully developed [7].

Definitive diagnosis is obtained by histology; however, preoperative biopsies may not establish a definitive diagnosis in most cases [3, 5]. Unlike leiomyosarcomas, leiomyomas display no cytologic atypia or necrosis, and mitotic activity is rarely demonstrated [8]. In contrast to GIST, leiomyomas are uniformly negative for CD117 and CD34 and are positive for muscle markers including smooth muscle actin and desmin [1].

The treatment of choice is complete surgical resection, which is both diagnostic and therapeutic. The aim of surgery is to obtain negative margins of resection preserving function [7]. The local recurrence after resection of perineal leiomyomas is rare.

## 6. Conclusion

Giant perineal leiomyomas are uncommon tumors that can grow to large dimensions and are usually seen in young female patients. Imaging is helpful in characterizing and determining the extent of these tumors. Complete resection is recommended and the local recurrence is rare.

## Figures and Tables

**Table 1:** Differential diagnosis for perineal masses [1, 3, 5, 7].

Differential diagnosis: perineal mass	
Soft tissue masses:	
leiomyosarcoma	
leiomyoma	
aggressive angiomyxoma	
sacrococcygeal teratoma	
liposarcoma	
solitary fibrous tumor	
Anorectal masses:	
perirectal abscess	
rectal duplication cyst	
rectal GIST	
anal squamous cell carcinomas	
anal adenocarcinomas	
Genitourinary masses:	
urethral cancer	
transitional cell carcinoma	
squamous cell carcinoma	
adenocarcinoma	
pedunculated uterine leiomyoma	
complex Bartholin mass	

Metastatic disease	

**Table 2:** Comparative table showing perineal leiomyoma case reports.

	Perineal leiomyoma case reports								
Author	Roy et al.	Agostini et al.	Lombana et al.	Brox-Jimenez et al.	Shuch et al.	Bernal-Sprekelsen et al.	Koc et al.	Oliveira-Brito et al.	Our patient
Year	1998	2006	2007	2007	2007	2010	2010	2011	2011
Country	India [11]	Italy [6]	Colombia [3]	Spain [4]	USA [8]	Spain [5]	Turkey [1]	Brazil [2]	USA
Age	47	56	37	48	60	30	47	36	41
Sex	F	F	F	F	F	F	F	F	F
Size	6.0 × 6.0 cm	22.0 × 13.0 cm	10.0 × 8.0 cm	6.0 × 7.0 cm	4.0 × 4.0 × 3.0 cm	7.4 × 5.1 × 5.4 cm	6.5 × 4.0 × 2.3 cm	15.0 × 6.5 × 7.5 cm	24.0 × 12.0 × 8.0 cm
SMA	nd	POS	nd	nd	POS	POS	POS	POS	POS
Desmin	nd	nd	nd	nd	POS	nd	POS	POS	POS
CD 117	nd	nd	nd	nd	nd	nd	NEG	NEG	NEG
CD 34	nd	nd	NEG	nd	nd	nd	NEG	NEG	NEG
S100	nd	nd	nd	nd	nd	nd	NEG	NEG	NEG
Surgery	WE	WE	WE	WE	WE	WE	WE	WE	WE
Margins	R0	R0	R0	R0	R0	R0	R0	R0	R0
Nuclear pleomorphism	NR	NEG	NR	NR	NEG	NR	NR	NEG	NEG
Necrosis	NR	NEG	POS	NEG	NEG	NEG	NR	NEG	NEG
Mitotic activity	<5/10	NEG	NEG	<5/10	NEG	NEG	NEG	NEG	<5/10
Atypia	NEG	NEG	NEG	NEG	NEG	NEG	NEG	NEG	POS
Uterine leiomyoma	Y	Y	NR	Y	Y	NR	NR	NR	Y
Recurrence	NONE	NONE	NR	NONE	NR	NONE	NONE	NR	NONE
Followup	24 m	16 m	NR	16 m	NR	14 m	12 m	NR	24 m

F: female; NEG: negative; POS: positive; nd: not done; NR: not reported; R0: negative margins; WE: wide excision; Y: yes; SMA: smooth muscle actin.

**Table 3:** MRI studies in perineal leiomyomas.

Author	MRI studies in perineal leiomyoma cases
Agostini et al. 2006	T1 hypointense
Italy [6]	T2 hypointense Solid mass multilobulated

Shuch et al. 2007	T1 heterogenous isointensity
USA [8]	T2 hyper- and hypointensity with areas of enhancement

Koc et al. 2010	T1 homogenously hyperintense
Turkey [1]	T2 heterogeneously hyperintense with significant but heterogeneous enhancement

**Our patient 2011**	**T1 midrange with diminished signal at its inferior portion**
**USA**	**T2 heterogeneously increasing signal in the inferior portion with diffuse enhancement**

MRI: magnetic resonance imaging.
